# Racial and Sex Inequities in the Use of and Outcomes After Left Ventricular Assist Device Implantation Among Medicare Beneficiaries

**DOI:** 10.1001/jamanetworkopen.2022.23080

**Published:** 2022-07-27

**Authors:** Thomas M. Cascino, Sriram Somanchi, Monica Colvin, Grace S. Chung, Alexander A. Brescia, Michael Pienta, Michael P. Thompson, James W. Stewart, Devraj Sukul, Daphne C. Watkins, Francis D. Pagani, Donald S. Likosky, Keith D. Aaronson, Jeffrey S. McCullough

**Affiliations:** 1 University of Michigan, Division of Cardiovascular Disease, Ann Arbor; 2 University of Notre Dame, Mendoza College of Business, Department of IT Analytics and Operations, Notre Dame, Indiana; 3 University of Michigan School of Public Health, Department of Health Management and Policy, Ann Arbor; 4 University of Michigan, Department of Cardiac Surgery, Ann Arbor; 5 University of Michigan, School of Social Work, Ann Arbor

## Abstract

**Importance:**

While left ventricular assist devices (LVADs) increase survival for patients with advanced heart failure (HF), racial and sex access and outcome inequities remain and are poorly understood.

**Objectives:**

To assess risk-adjusted inequities in access and outcomes for both Black and female patients and to examine heterogeneity in treatment decisions among patients for whom clinician discretion has a more prominent role.

**Design, Setting, and Participants:**

This retrospective cohort study of 12 310 Medicare beneficiaries used 100% Medicare Fee-for-Service administrative claims. Included patients had been admitted for heart failure from 2008 to 2014. Data were collected from July 2007 to December 2015 and analyzed from August 23, 2020, to May 15, 2022.

**Exposures:**

Beneficiary race and sex.

**Main Outcomes and Measures:**

The propensity for LVAD implantation was based on clinical risk factors from the 6 months preceding HF admission using XGBoost and the synthetic minority oversampling technique. Beneficiaries with a 5% or greater probability of receiving an LVAD were included. Logistic regression models were estimated to measure associations of race and sex with LVAD receipt adjusting for clinical characteristics and social determinants of health (eg, distance from LVAD center, Medicare low-income subsidy, neighborhood deprivation). Next, 1-year mortality after LVAD was examined.

**Results:**

The analytic sample included 12 310 beneficiaries, of whom 22.9% (n = 2819) were Black and 23.7% (n = 2920) were women. In multivariable models, Black beneficiaries were 3.0% (0.2% to 5.8%) less likely to receive LVAD than White beneficiaries, and women were 7.9% (5.6% to 10.2%) less likely to receive LVAD than men. Individual poverty and worse neighborhood deprivation were associated with reduced use, 2.9% (0.4% to 5.3%) and 6.7% (2.9% to 10.5%), respectively, but these measures did little to explain observed disparities. The racial disparity was concentrated among patients with a low propensity score (propensity score <0.52). One-year survival by race and sex were similar on average, but Black patients with a low propensity score experienced improved survival (7.2% [95% CI, 0.9% to 13.5%]).

**Conclusions and Relevance:**

In this cohort study of Medicare beneficiaries hospitalized for HF, disparities in LVAD use by race and sex existed and were not explained by clinical characteristics or social determinants of health. The treatment and post-LVAD survival by race were equivalent among the most obvious LVAD candidates. However, there was differential use and outcomes among less clear-cut LVAD candidates, with lower use but improved survival among Black patients. Inequity in LVAD access may have resulted from differences in clinician decision-making because of systemic racism and discrimination, implicit bias, or patient preference.

## Key Points

**Question:**

How large are racial and sex inequities in left ventricular assist device (LVAD) use and outcomes?

**Findings:**

In this cohort study of 12 310 Medicare beneficiaries with heart failure at risk for requiring an LVAD, Black beneficiaries were 3.0% less likely than White beneficiaries to receive an LVAD, and female beneficiaries were 7.9% less likely than male patients to receive LVAD therapy. One-year survival among groups was similar after adjusting for individual poverty and community-level social determinants of health.

**Meaning:**

These findings suggest that there is less aggressive use of LVADs for Black and female Medicare beneficiaries, likely resulting from differences in clinician decision-making because of systemic racism and discrimination, implicit bias, or patient preference.

## Teaser

This cohort study assesses inequities in access and outcomes in the receipt of left ventricular assist device (LVAD) therapy for Black and female patients in the US.

## Introduction

Heart failure (HF) prevalence continues to rise with disproportionate increases among women and Black Americans.^1^ Among adults 65 years or older, there is an approximately 50% increase in age-adjusted HF mortality among Black men and women compared with White men and women.^2^ Left ventricular assist device (LVAD) use has become an increasingly viable option with more than 3000 patients receiving an LVAD per year^3^ and a 1-year survival similar to heart transplant.^4^ Historically, women and Black patients have been less likely to receive advanced HF therapies, including LVADs.^5,6,7,8^

Recent work suggests population-level increases in durable LVAD use among women^6^ and Black patients.^5,9^ Given the higher prevalence and worse HF outcomes, LVADs may still be underused within these populations as prior work has been limited by an understanding of the number of patients with advanced HF who may be eligible for LVAD therapy.^5^ Additionally, the reasons for such inequities remain uncertain. Recent research has sought to understand how structural racism and discrimination^10^—the macro-level conditions, such as institutional policies that limit opportunities and resources based on race, ethnicity, sex, or socioeconomic status^11^—and HF clinicians' implicit biases may contribute.^12^ As such, an understanding of the severity and potential drivers of inequities is necessary to improve equitable LVAD access.

This retrospective cohort study of Medicare patients hospitalized for HF was conducted to quantify sex and racial inequities in LVAD therapy use and outcomes. It was hypothesized that inequities in treatment access and outcomes for both Black and female patients would exist after adjusting for individual patient comorbidities and social determinants of health (SDOH), and there would be heterogeneity in inequities with the greatest disparities where treatment decisions are less clear and clinician discretion plays a more prominent role.

## Methods

This cohort study was approved by the University of Michigan institutional review board and followed the Strengthening the Reporting of Observational Studies in Epidemiology (STROBE) reporting guideline. Data were collected from July 2007 to December 2015 and analyzed from August 23, 2020, to May 15, 2022.

### Data Source and Study Population

The data included 100% Medicare Fee-for-Service (FFS) administrative claims (Parts A and B) from July 2007 through December 2015. Medicare claims were selected because (1) all beneficiaries have insurance with inclusive clinician networks, eliminating selection bias because of insurers’ networks and prices; (2) demographic data including race are systematically collected; and (3) patients can be tracked longitudinally.

The study population included all Medicare FFS beneficiaries hospitalized with systolic HF between 2008 to 2014 with at least 6 months of continuous enrollment preceding hospitalization. Patients with systolic HF were identified using *International Classification of Diseases, Ninth Revision (ICD-9)* codes 428.30, 428.31, 428.32, and 428.33 or receipt of LVAD. Patients whose race was other than Black or White were excluded because of small sample sizes. Race was self-identifiable, and ethnicity was not specifically considered in this study because race and ethnicity are recorded in Medicare data as a single variable.

### Outcomes

The primary outcomes of receipt of LVAD and 1-year mortality and the secondary outcome of 30-day readmissions were assessed to determine associations between race, sex, and outcomes. LVAD receipt was evaluated during the index hospitalization using *ICD-9* procedure codes 37.66 in combination with diagnosis related group 001 or 002, excluding explants.

### Primary Estimators and Social Determinants of Health

The primary independent estimators were race (ie, Black or White) and sex (ie, male or female). Recognizing that race is a sociopolitical framework rather than biological,^13^ race was chosen as a primary estimator to begin understanding the potential association of structural racism and discrimination on access to LVAD therapies.

SDOH are conditions in which people live and work that impact health outcomes.^14^ The associations of neighborhood and individual socioeconomic status were explored to understand how restrictions in social and economic opportunities affect patients with HF. Neighborhood socioeconomic status was captured using the Social Deprivation Index (SDI).^15,16^ SDI was transformed to a scale from 0 to 1. Individual poverty was measured using Medicare Part D’s Low-Income Subsidy (LIS) status (income <150% of Federal Poverty Level).^17^

### Statistical Analysis

The goal was to measure whether race and sex were associated with LVAD receipt and post-LVAD survival after adjusting for neighborhood- and individual-level SDOH. The analysis had 3 steps: (1) identify the subset of patients at risk of needing an LVAD; (2) measure the associations of race and sex on LVAD receipt; and (3) model the associations of race and sex on 1-year survival for LVAD patients.

#### Sample Selection

We first estimated patient-specific propensities to receive LVAD treatment. These estimates were based on a high-dimensional set of patient characteristics described in eTables 1 and 2 in the Supplement. This was necessary because LVAD therapy is not relevant to most patients with HF. The information captured included demographics (excluding race and sex), health care use for the past 6 months, comorbidities present on or before the index admission, and interactions between these variables. This analysis faced 3 challenges: (1) there are many patient characteristics; (2) the proper specification is unknown; and (3) there is a large class imbalance (ie, LVADs are rare).

Treatment and mortality probabilities were estimated using nonparametric methods. Our approach used XGBoost for flexible (ie, nonparametric) prediction combined with synthetic minority oversampling (SMOTE) to address the class imbalance. Given that LVADs are rare, SMOTE helps to improve predictive accuracy. SMOTE generates a synthetic sample that oversamples the rare outcome (ie, LVAD) and undersamples the more prevalent outcome (ie, medical management) to improve predictive accuracy. Predictions are generated by the XGBoost algorithm to capture all nonlinear combinations of the risk factors for the SMOTE-generated samples. Models were cross-validated for out-of-sample predictive accuracy. Further details are provided in eMethods 1 in the Supplement. Observations were eliminated if LVAD propensity was less than 0.05 because no patients with a propensity below 0.05 received an LVAD. For patients with multiple hospitalizations, beneficiaries were included during the admission with the highest propensity to receive an LVAD.

#### LVAD Treatment Use

A series of models were used to measure associations between LVAD receipt and patients’ race and sex (Model 1). This initial model was followed by cumulative adjustments for estimated patient LVAD propensity (Model 2), patient age (Model 3), proximity to LVAD centers (Model 4), patient’s LIS status (Model 5), SDI (Model 6), and allowing for the patient neighborhood (ie, zip code) random effects (Model 7). Models 5 through 7 were estimated on the Part-D beneficiary subsample. Interactions were included to allow for parameter heterogeneity. All models included interactions between race and sex. Furthermore, patient race and sex were interacted with the LVAD propensity, patient age, and age squared. Each model was estimated by logistic regression. Detailed specifications are described in eMethods 2 in the Supplement. Results are presented as marginal effects (ie, the associated percentage increase or decrease in LVAD use).

#### LVAD Outcomes

Our models used logistic regression to examine post-LVAD survival differences by patient race and sex (Model 1). The models cumulatively adjusted for disease severity (Model 2), LVAD propensity (Model 3), hospital fixed-effects (Model 4), age (Model 5), proximity to the nearest LVAD center (model 6), LIS status (Model 7), SDI (Model 8), and a specification where hospital fixed effects are excluded and patient neighborhood (ie, zip code) random effects are included (Model 9). One-year survival was predicted for LVAD recipients to measure disease severity using the same independent variables used to estimate the LVAD propensity. The model is estimated using an XGBoost algorithm with 10-fold cross-validation to select the hyperparameters that improve for out-of-sample predictive accuracy (eMethods 1 in the Supplement). The hospital-specific associations in Models 4 to 8 allow for unobserved quality or severity differences correlated with individual hospitals. Models 7 to 9 were estimated on the Part D subsample. This same approach was applied to the secondary outcome of 30-day readmissions. A post hoc analysis was performed comparing the association between race and 1-year survival after LVAD for recipients with a propensity above and below 0.52 after finding reduced LVAD use at a propensity less than this cutoff. Results were reported as marginal effects. Detailed specifications of all models are described in eMethods 2 in the Supplement. *P* ≤ .05 was considered statistically significant using 2-sided tests of significance. SMOTE and XGBoost were implemented using R version 4.0.5 (R Project for Statistical Computing), and all other analyses were conducted using Stata/MP version 17.0 (StataCorp).

## Results

Our initial sample included 311 265 Medicare fee-for-service beneficiaries with an inpatient admission and primary diagnosis of systolic HF. We identified 12 310 beneficiaries with an LVAD use probability of at least 0.05. Table 1 presents the characteristics of patients by race. Of the 12 310 beneficiaries, 2819 (22.9%) were Black patients, and 2920 (23.7%) were female patients. Compared with White patients, Black patients were younger (mean [SD], 56.3 [12.3] years vs 64.8 [10.3] years). The subset of the sample with Medicare Part D data used to identify those with LIS status included 1934 of 7711 Black beneficiaries (25.1%). Among this subset, Black patients compared with White patients had a higher mean (SD) SDI (0.70 [0.25] vs 0.45 [0.26]) and had a greater prevalence of LIS (1885 [75.3%] vs 2435 [35.5%]).

**Table 1.** Characteristics of Patients by Race

Characteristics	Patients, No. (%)		
	Full Sample (N = 12 310)	Black (n = 2819)	White (n = 9491)
LVAD receipt	5909 (48.0)	1356 (48.1)	4556 (48.0)
LVAD propensity, mean (SD)	0.53 (0.50)	0.51 (0.50)	0.54 (0.50)
Survival, predicted, mean (SD)	0.73 (0.19)	0.76 (0.18)	0.72 (0.19)
Demographic characteristics			
Patient age, mean (SD), y	62.8 (11.3)	56.3 (12.3)	64.8 (10.3)
Sex			
Female	2917 (23.7)	1020 (36.2)	1898 (20.0)
Male	9393 (76.3)	1799 (63.8)	7593 (80.0)
Social deprivation index, mean (SD)	0.51 (0.28)	0.70 (0.25)	0.45 (0.26)
Miles to LVAD hospital, mean (SD)	81.9 (67.3)	63.5 (62.1)	87.4 (67.8)
Low income subsidy^a^	4259 (45.5)	1885 (75.3)	2435 (35.5)
Clinical characteristics			
Hypertension	2302 (18.7)	730 (25.9)	1576 (16.6)
Diabetes	5429 (44.1)	1235 (43.8)	4195 (44.2)
Kidney failure	7706 (62.6)	1990 (70.6)	5714 (60.2)
COPD	2954 (24.0)	578 (20.5)	2373 (25.0)
Hyponatremia	2290 (18.6)	488 (17.3)	1803 (19.0)
Thyroid conditions	2191 (17.8)	400 (14.2)	1794 (18.9)
Cancer, any	825 (6.7)	127 (4.5)	693 (7.3)

Abbreviations: COPD, chronic obstructive pulmonary disease; LVAD, left ventricular assist device; y, years.

^a^ Sample sizes are smaller for the low-income subsidy (full sample, N = 7711; Black patients, n = 1934; White, n = 5777).

**Figure 1.**
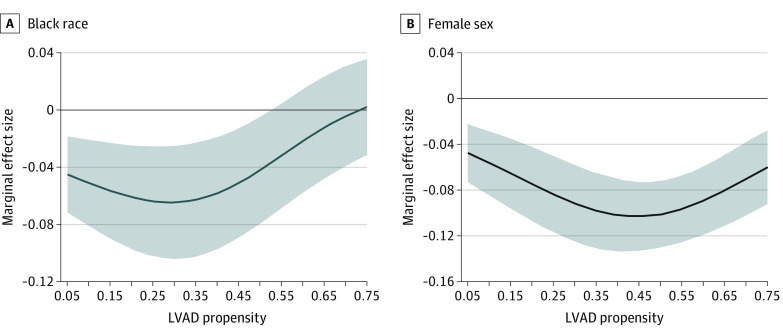
Associations of Black Race and Female Sex With LVAD Use The marginal effect size with 95% CIs of (A) race and (B) sex on the use of LVAD conditional on clinical risk, age, distance to hospital, individual socioeconomic status, and neighborhood effects. Compared with White patients, Black beneficiaries at a lower propensity were less likely to receive an LVAD when hospitalized for systolic heart failure. LVAD use in women was less than men across the spectrum of LVAD propensity. LVAD indicates left ventricular assist devices.

LVAD use was higher among White and male patients. These differences were maintained across the LVAD propensity distributions (eFigure 1 in the Supplement). The marginal effect sizes of Black race and female sex were –1.7% (95% CI, –3.9% to 0.5%) and –12.5% (–14.6% to –10.4%), respectively (Table 2). Race and sex were associated with a 3% (95% CI, 0.2% to 5.8%) and 7.9% (95% CI, 5.6% to 10.2%) decreased LVAD use, respectively, after the inclusion of clinical characteristics, distance from a center, individual poverty, and neighborhood-level social deprivation (Figure 1). The inclusion of neighborhood random effects did not change these associations (Model 7). These differences were not statistically significant for high-propensity (≥0.52) Black patients (Figure 1). The disparity became large (about 5%) and statistically significant for lower propensity patients (<0.52), which represents 1155 (41%) of Black patients with HF. On average, Black men were 6.0% (95% CI, 2.5% to 9.5%) less likely to receive an LVAD than White men, with greater differences at lower propensities (eFigure 2 in the Supplement). There was no difference in receipt of LVAD among Black females compared to White females at any propensity (1.3%; 95% CI, −2.8% to 5.6%).

**Table 2.** Association of Patient Characteristics with LVAD Use^a^

Variables	Marginal effect size (95% CI)						
	Model 1	Model 2	Model 3	Model 4	Model 5	Model 6	Model 7
Black race (vs White)	–0.017 (–0.039 to 0.005)	–0.022 (–0.041 to –0.003)	–0.036 (–0.057 to –0.015)	–0.043 (–0.065 to –0.022)	–0.045 (–0.072 to –0.018)	–0.030 (–0.058 to –0.002)	–0.030 (–0.058 to –0.002)
Female (vs male)	–0.125 (–0.146 to –0.104)	–0.089 (–0.108 to –0.070)	–0.090 (–0.109 to –0.071)	–0.090 (–0.109 to –0.071)	–0.079 (–0.102 to –0.056)	–0.079 (–0.102 to –0.056)	–0.079 (–0.102 to –0.056)
LVAD propensity	NA	0.965 (0.941 to 0.989)	0.962 (0.938 to 0.986)	0.960 (0.936 to 0.984)	0.956 (0.926 to 0.986)	0.953 (0.923 to 0.983)	0.954 (0.924 to 0.984)
Age, per year	NA	NA	–0.001 (–0.002 to 0.0003)	–0.001 (–0.0013 to 0.0001)	–0.002 (–0.003 to –0.001)	–0.002 (–0.003 to –0.001)	–0.002 (–0.003 to –0.001)
Distance to LVAD Hospital, per 10 miles	NA	NA	NA	–0.002 (–0.003 to –0.001)	–0.003 (–0.004 to –0.002)	–0.003 (–0.004 to –0.002)	–0.003 (–0.004 to –0.002)
Low-income subsidy	NA	NA	NA	NA	–0.036 (–0.060 to –0.012)	–0.029 (–0.053 to –0.005)	–0.030 (–0.054 to –0.005)
Social deprivation index	NA	NA	NA	NA	NA	–0.067 (–0.105 to –0.029)	–0.068 (–0.106 to –0.030)
Observations	12 310	12 310	12 310	12 310	7711	7710	7710
Year indicators	Yes	Yes	Yes	Yes	Yes	Yes	Yes
Neighborhood RE	No	No	No	No	No	No	Yes
Part D enrollees only	No	No	No	No	Yes	Yes	Yes

Abbreviations: LVAD, left ventricular assist device; NA, not applicable; RE, random effect.

^a^ Model 1 was conditional on age, sex, interactions of age and sex, and year indicators. After adjusting for expected LVAD use based on patient characteristics (Model 2), incorporating interactions with patient age (Model 3), distance to LVAD centers (Model 4), individual poverty (Model 5), and neighborhood-level social deprivation (Model 6), Black race and female sex were associated with a –3.0 (95% CI, –5.8 to –0.2) and –7.9 (95% CI, –10.2 to –5.6) percentage points decrease use of LVAD. These associations were consistent when neighborhood included as a random effect (Model 7).

**Figure 2.**
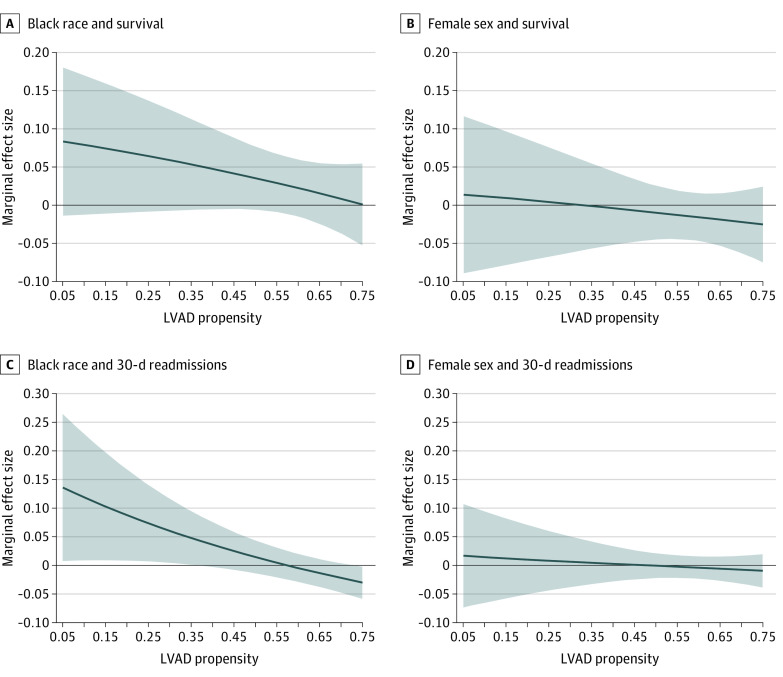
Association of Race and Sex With 30-Day Readmissions by LVAD Propensity The posterior estimations of the marginal effect size with 95% CIs of race and sex on 1-year survival after LVAD adjusted for clinical risk, distance to hospital, individual socioeconomic status, and neighborhood effects. Compared with White patients, Black beneficiaries at a lower propensity to receive an LVAD had a trend toward improved survival in the year after LVAD (A). Women had similar survival across all LVAD propensity (B). Compared with White patients, Black beneficiaries at a lower propensity to receive an LVAD had increased 30-day readmissions after LVAD (C). Women had similar 30-day readmissions across all LVAD propensity (B). LVAD indicates left ventricular assist devices.

The 1-year survival rates for Black and White LVAD recipients were 76% and 72%. Male and female patients had equivalent survival rates of 72% and 73%, respectively. Table 3 presents logistic regression results for 1-year survival after LVAD. Mean survival rates for Black and female patients are nearly identical to their White and male counterparts after controlling for disease severity (Models 2-9 in Table 3). There was no significant interaction between expected survival and either race or sex and actual 1-year survival (eFigure 3 in the Supplement). The differences in survival in Black recipients compared with White recipients decreased with increasing LVAD propensity (Figure 2). In the post hoc analysis, 1-year survival rates for low propensity Black and White recipients were 84.4% and 77.0%. Black patients with LVAD propensities less than 0.52 (26.3% of Black LVAD recipients) had significantly higher 1-year survival rates than White LVAD patients (difference, 7.2% [95% CI, 0.9-13.5]). These findings were robust to controls for disease severity, age, proximity to VAD centers, individual poverty, SDI, and hospital fixed effects. Findings for 30-day readmissions showed a meaningful increase in 30-day readmissions for Black recipients with low propensity compared with White patients after LVAD (eTable 3 in the Supplement and Figure 2).

**Table 3.** Association of Patient Characteristics with 1-Year Survival Conditional Upon LVAD Use^a^

Variables	Marginal effect size (95% CI)								
	Model 1	Model 2	Model 3	Model 4	Model 5	Model 6	Model 7	Model 8	Model 9
Black race (vs White race)	0.039 (0.013 to 0.066)	0.010 (–0.015 to 0.034)	0.011 (–0.014 to 0.036)	0.006 (–0.001 to 0.013)	0.010 (–0.005 to 0.025)	0.009 (–0.005 to 0.024)	0.008 (–0.013 to 0.028)	0.007 (–0.014 to 0.027)	0.005 (–0.034 to 0.043)
Female (vs male)	–0.006 (–0.035 to 0.023)	–0.011 (–0.037 to 0.016)	–0.011 (–0.037 to 0.016)	0.001 (–0.007 to 0.009)	–0.0001 (–0.013 to 0.013)	–0.0002 (–0.013 to 0.013)	0.002 (–0.014 to 0.018)	0.002 (–0.014 to 0.018)	–0.015 (–0.046 to 0.016)
Survival, predicted	NA	0.830 (0.790 to 0.870)	0.829 (0.788 to 0.870)	0.155 (0.104 to 0.206)	0.271 (–0.062 to 0.604)	0.275 (–0.062 to 0.612)	0.272 (–0.157 to 0.701)	0.267 (–0.158 to 0.692)	0.754 (0.702 to 0.806)
VAD propensity	NA	NA	–0.015 (–0.082 to 0.053)	–0.007 (–0.022 to 0.008)	–0.015 (–0.047 to 0.018)	–0.015 (–0.048 to 0.018)	–0.023 (–0.076 to 0.030)	–0.022 (–0.074 to 0.030)	–0.029 (–0.113 to 0.055)
Age, per year	NA	NA	NA	NA	–0.0002 (–0.001 to 0.0003)	–0.0002 (–0.001 to 0.0003)	–0.0003 (–0.001 to 0.0004)	–0.0003 (–0.001 to 0.0004)	–0.001 (–0.002 to 0.001)
Distance to LVAD Hospital, per 10 miles	NA	NA	NA	NA	NA	–0.0003 (–0.001 to 0.0003)	0.000 (–0.001 to 0.001)	0.000 (–0.001 to 0.001)	0.000 (–0.002 to 0.002)
Low-income subsidy	NA	NA	NA	NA	NA	NA	0.003 (–0.009 to 0.015)	0.003 (–0.009 to 0.014)	0.006 (–0.025 to 0.037)
Social deprivation index	NA	NA	NA	NA	NA	NA	NA	0.004 (–0.014 to 0.021)	0.017 (–0.029 to 0.063)
Observations	6576	6576	6576	6490	6490	6490	4032	4032	4099
Hospital FE	No	No	No	Yes	Yes	Yes	Yes	Yes	No
Year indicators	Yes	Yes	Yes	Yes	Yes	Yes	Yes	Yes	Yes
Neighborhood RE	No	No	No	No	No	No	No	No	Yes
Part D enrollees only	No	No	No	No	No	No	Yes	Yes	Yes

Abbreviations: FE, fixed effect; LVAD, left ventricular assist device; NA, not applicable; RE, random effect; SDI, social deprivation index.

^a^ Model 1 was conditional on age, sex, interactions of age and sex, and year indicators. Survival by race and sex was similar after adjusting for clinical risk (Model 2), LVAD propensity (Model 3), hospital fixed-effect (Model 4), age (Model 5), distance to hospital (Model 6), LIS (Model 7), SDI (Model 8), and neighborhood random effects (Model 9).

## Discussion

Our study identified racial and sex disparities in LVAD therapy use. Black and female beneficiaries were less likely than their White and male counterparts to receive LVAD therapy. Individual poverty and community-level SDOH were associated with lower LVAD treatment rates, but racial and sex disparities were robust to controlling for these factors. Finally, conditional 1-year survival rates for LVAD recipients were equal for female patients and at least as high for Black patients compared with male and White recipients. In particular, Black patients with a lower propensity to receive LVAD treatment had higher survival rates than White patients with a similar propensity to receive an LVAD.

Recent work has demonstrated expanding the use of LVADs among Black and female patients.^5,6,9^ Given the higher prevalence of HF among racial and ethnic minorities^1^ and increased HF mortality,^2^ it is uncertain whether the rising use of LVADs is proportional to the number of Black patients with HF.^18^ For women, population-level data continues to show underuse of LVADs, with mixed associations with outcomes compared with men after LVAD.^6,7,8,19,20^ The current study further elucidates the relationship between LVAD use and patient race and sex.

LVAD use among women was low for Black and White women relative to men. Our findings are consistent with work demonstrating sex bias impacts therapeutic decisions for cardiovascular care^21^ and results in inequities in LVAD use, with women representing only approximately 20% of LVAD recipients.^3,6^ Women in our study received LVAD therapy at a lower rate across the distributions of propensity and severity. While the treatment disparities were more prominent for women than for Black patients, there was no observed variation in the potential underlying mechanisms. Approaches toward addressing sex inequities may include prioritizing adequate representation in clinical trials among those designing^22^ and enrolling^23^ and the funding of mixed methods research aimed at understanding reasons for and methods to address sex inequality.^24^

In contrast to sex disparities, racial disparities in access are unevenly distributed across patients’ clinical characteristics. The underuse of LVADs appears predominantly in Black men with lower probabilities of LVAD receipt.^5^ There was almost no racial disparity among patients with high LVAD propensities, but disparities were large and significant among patients with below-median propensities.

The distribution of survival disparities across the LVAD propensities becomes critical to interpreting these results. Observed LVAD survival is significantly higher even with increased readmissions for low-propensity Black LVAD recipients. Higher HF readmissions without association to mortality for Black patients despite similar clinical characteristics is a well-described phenomenon.^25,26,27,28,29,30^ The literature supports that the readmissions are most likely related to the SDOH (eg, health literacy, social support) and not HF severity or the health care system.^26,31^ Alternatively, unobserved severity that increases readmissions without impacting mortality could explain the findings.

While high survival rates are the goal, the decreased use with improved survival suggests treatment is less aggressive for Black patients who are similarly ill and have a lower propensity. This pattern has several potential interpretations because a higher propensity score seeks to identify a patient who would be an ideal LVAD recipient (eg, advanced HF without contraindications), and a lower propensity score suggests a less ideal candidate (eg, less severe HF). Clinicians observe many factors not present in our data, and treatment could be based on unobserved severity or contraindications. Conversely, physicians or patients may have biased beliefs or preferences. Given the high mortality for LVAD candidates who receive medical therapy (ie, approximately 50% survival at 2 years),^32^ low use among ambulatory patients with HF in the Intermacs registry,^3^ and the historical context of racial health inequities in the US,^33^ these findings are most consistent with a pattern of structural racism and discrimination.

Finally, the association of both neighborhood and individual poverty on LVAD use and outcomes merits discussion. The association between race and decreased LVAD use persisted, although it was reduced after adjusting for individual socioeconomic status and neighborhood effects. Disproportionate differences by race in socioeconomic status and neighborhood deprivation^31,34,35^ and reduced LVAD access for low socioeconomic status patients with HF are consistent with prior work.^36,37^ These differences have resulted from systemic racism^33^ and are associated with inequitable LVAD access despite no association with worse survival after LVAD. With the large racial disparities in wealth,^38^ economically disadvantaged racial and ethnic minorities may be systematically withheld LVADs.

For clinicians, the finding of reduced LVAD use for Black patients suggests that implicit biases or personally mediated racism impact decision-making. Both implicit biases, which refer to the unconscious attitudes that impact our actions,^18^ and personally mediated racism, referring to conscious or unconscious discrimination in the form of differential actions according to race,^39^ have been shown to influence the quality of care. Since Schulman et al^21^ showed differential management of chest pain based on race and sex, several examples of differential treatment based on race and sex have been illustrated among patients with HF,^40,41^ including decision-making around advanced HF therapies.^12^ While the underuse of LVAD among Black patients may result from unmeasured patient characteristics, the robust and consistent findings of prior work suggest that inequities in use result from either implicit biases or structural racism. Addressing these issues to improve equity will require a multifaceted approach. In the short term, strategies could include addressing clinician bias with implicit bias training, creating evaluation algorithms with protocols that do not include subjective assessments to the extent possible and removing information suggesting race, ethnicity, and sex from discussion at multidisciplinary meetings.^12,42^ Longer-term approaches that show promise include revamping the training of health professionals to ensure education on structural racism is a core component of the curriculum^33^ and diversifying the health care workforce.^43^

### Limitations

This study has limitations, and several are inherent to using Medicare claims data. Ideally, one could observe the treatment effect of LVAD relative to medical therapy for each patient. This is impossible because claims data do not capture many important patient characteristics. For example, it is possible that lower treatment rates among Black patients with low propensity are appropriate because of higher unobserved illness severity for White patients—factors that may not be captured in claims data that would be observed by patients and clinicians. The result is unlikely driven by Black patients having lower unobserved HF risks given the higher prevalence^1^ and previous research, including data with more clinical detail, suggesting increased clinical severity among Black patients with HF.^18^ It is also possible that the improved outcomes are a result of Black recipients being less sick at the time of implant. This is unlikely because there is lower LVAD receipt for Black patients who are less sick compared with White patients.^5^ The distinction between clinician and patient preferences is another essential consideration. Our data cannot distinguish between the actions of patients and clinicians although LVAD preferences are similar across patient race and sex.^44^ Lastly, our results might also be biased by unobserved neighborhood differences. Our SDI metrics, for example, are based on 5-digit zip codes. Census-tract level SDI measures, which cannot be matched to our data, might be more accurate. Our analysis suggests that such bias would be minimal because we control for individual-level poverty and the SDI parameters are robust to the inclusion of zip code random effects.

## Conclusions

The findings of this cohort study suggest that disparities in LVAD use by race and sex are not entirely explained by clinical characteristics, distance, individual socioeconomic status, or neighborhood deprivation. It will be critical for future research to advance evaluations of interventions at the clinician-level (eg, implicit bias training) to address persistent inequities in access and outcomes for LVAD patients.

## Notes

Supplement. **eTable 1.** Definition of Variables Used in the LVAD and Mortality Prediction Models **eTable 2. **Betos Codes Based on Part B Claims **eTable 3. **Marginal Effect Sizes for 30-Day Readmissions Following Discharge From LVAD **eMethods 1. **Sample Selection and LVAD Propensity Estimation **eMethods 2. **Empirical Methods **eFigure 1.** Unconditional Effects of Race and Sex on LVAD Use **eFigure 2.** Marginal Effect Sizes of Black Race by Sex on LVAD Use **eFigure 3. **Incremental Effect of Race and Sex on Survival by Expected Survival
