# Supplementary material for: Racial and Sex Inequities in the Use of and Outcomes After Left Ventricular Assist Device Implantation Among Medicare Beneficiaries
Source: JAMA Netw Open. 2022 Jul 27;5(7):e2223080. doi: 10.1001/jamanetworkopen.2022.23080 (PMC9331085; doi:10.1001/jamanetworkopen.2022.23080)
Supplement: Supplement. — eTable 1. Definition of Variables Used in the LVAD and Mortality Prediction Models eTable 2. Betos Codes Based on Part B Claims eTable 3. Marginal Effect Sizes for 30-Day Readmissions Following Discharge From LVAD eMethods 1. Sample Selection and LVAD Propensity Estimation eMethods 2. Empirical Methods eFigure 1. Unconditional Effects of Race and Sex on LVAD Use eFigure 2. Marginal Effect Sizes of Black Race by Sex on LVAD Use eFigure 3. Incremental Effect of Race and Sex on Survival by Expected Survival [file jamanetwopen-e2223080-s001.pdf]

Supplemental Online Content

Cascino TM, Somanchi S, Colvin M, et al. Racial and sex inequities in the use of and outcomes after left ventricular assist device implantation among Medicare beneficiaries. *JAMA Netw Open*. 2022;5(7):e2223080. doi:10.1001/jamanetworkopen.2022.23080

eTable 1. Definition of Variables Used in the LVAD and Mortality Prediction Models

eTable 2. Betos Codes Based on Part B Claims

eTable 3. Marginal Effects for 30-Day Readmissions Following Discharge From LVAD

eMethods 1. Sample Selection and LVAD Propensity Estimation

eMethods 2. Empirical Methods

eFigure 1. Unconditional Effects of Race and Sex on LVAD Use

eFigure 2. Marginal Effects of Black Race by Gender on LVAD Use

eFigure 3. Incremental Effect of Race and Gender on Survival by Expected Survival

This supplemental material has been provided by the authors to give readers additional information about their work.

© 2022 Cascino TM et al. *JAMA Network Open.*

eTable 1. Definition of Variables Used in the LVAD and Mortality Prediction Models

Name from code	Outpatient past 6 months	Inpatient past 6 months	Inpatient, current	ICD-9 Codes
Bleeding, GI	X		X	578*, 531, 531.2, 531.6, 532, 532.2, 532.4, 532.6, 533, 533.2, 533.4, 534.6, 534, 534.2, 534.4, 534.6, 535.1, 530.82, 456, 456.2, 569.3, 596.7
Bleeding, other	X			362.81, 459, 786.3
Bleeding, Tendency	X			286, 286.5, 286.6, 286.7, 286.9, 287.3, 287.4, 287.5, 287.8, 287.9, 289.9
CVD	X			430-432.99
CVD, Other				851/854.99
Renal Failure	X	X	X	404.01, 404.03, 404.11, 404.13, 404.91, 404.93
Renal insufficiency	X	X	X	584.00/586.99
Liver, Cirrhosis			X	570*, 571*, 572.2, 572.3, 572.4, 572.8
Liver, Hepatitis				070.2, 070.4, 070.6, 070.7
Peripheral Vascular Disease	X		X	443.9, 250.7, 443.8
Cancer, any	X		X	140.00/159.99, 160.00/165.99, 170.00/176.99, 179.00/184.99, 190.00/199.99, 200.00/209.99, 230.00/239.99, V58.0/V58.1
Ischemic Heart Disease	X	X	X	410*, 411*, 413.0*, 413.9*, 414.0*, 414.12, 414.2/414.49, 414.8/414.99
Rheumatic Heart Disease	X	X	X	393/398.99
Hypertension	X	X	X	352.11, 404/405.99
Other cardiovascular diagnoses	X			433.01, 433.11, 433.21, 433.31, 433.81, 433.91, 434*, 435.0*, 435.1*, 435.3*, 435.8*, 435.9*, 436*
Hypothyroid	X			402.01, 402.11, 402.91
Coronary Atherosclerosis	X			440*
Count of prior hospitalizations (6-month period)		X		
Diabetes	X	X	X	249*, 250*, 357.2, 362.01-362.06, 366.41
Heart Failure, Any	X	X		428*
Heart Failure, Systolic	X	X	X	428.2*, 428.4*
Heart Failure, Diastolic	X			428.3*
Thyroid diagnoses	X	X	X	240*-245*

© 2022 Cascino TM et al. *JAMA Network Open.*

Name from code	Outpatient past 6 months	Inpatient past 6 months	Inpatient, current	ICD-9 Codes
Malnutrition	X		X	262*, 263*
Anemia	X			280
Right and Left Cath		X		37.23
Right Cath		X	X	37.21
Arterial Cath		X	X	38.91, 38.93, 38.97/38.99
Alcohol and Drug Use	X	X	X	303.00/305.99, 305.1
COPD	X	X	X	490.00/492.99, 494*, 496*
Pneumonia	X	X	X	480.00/486.99
	X			295/298.99, V15.81, V60.0/V60.4
Fall-related Risk Factors	X		X	290.00/294.99, 331, 331.1, 333.4, 345*, 347, 458, 780.2, 780.3, E880/E888.9
Dementia	X			290*, 294/294.89, 331/331.29, 333.17, 797*
Dialysis, Venous			X	38.95
Dialysis			X	39.27, 39.42/39.43, 39.95
Other Vascular Procedures			X	39.00/39.26, 39.28/39.41, 39.44/39.64, 39.67/39.94, 39.96/39.99
			X	37.00/37.19, 37.3/37.49, 37.9/37.93, 37.98/37.99
			X	37.2, 37.24/37.29
Implantable Cardioverter Defibrillator			X	37.94/37.97
Pacemaker			X	37.7/37.89
Valve implant			X	35*
Mechanical Circ. Support			X	37.6, 37.62, 37.65, 37.61, 37.68, 37.67, 37.63/37.65, 39.65, 39.66
Hyponatremia	X	X	X	276.0*, 276.1*
Pulmonary Hypertension	X			416.0*
Tachycardia	X			785.0*
Oxygen Dependence	X			v46.1/V46.2
Other risk factors	X	X	X	016/016.06, 095.4*, 189.0*, 189.9*, 223.0*, 236.91, 249.4, 249.41, 250.4/250.43, 271.4, 283.11, 403.01, 403.11, 403.91, 442.1*, 580/588.99, 591*, 753.2*, 794.4*
Depression	X			296.2, 296.36, 300*

© 2022 Cascino TM et al. *JAMA Network Open.*

eTable 2. Betos Codes Based on Part B Claims

Variables	Betos and Specialty Code Values
Dialysis	P9A, P9B
Major Procedure, Cardiovascular, other	P2F
ECG	T2A
Echo, Heart	I3C
EKG monitor	T2C
Emergency Encounter	23
Psychiatric therapy	T
Cardiology	06, C3
Cardiac Electrophysiology	21
Diagnostic Radiology	30
Emergency Medicine	93
Internal Medicine	11
Infectious Disease	44
Clinical Laboratory (billing independently)	69
Nephrology	39
Oncology	83, 90, 91, 92, 93, 98
Ophthalmology	18
Pathology	22
Primary Care	01, 08, 16, 50
Podiatry	48
Psychiatry	26, 62, 68, 79, 86
Pulmonology	29
Cardiovascular surgery	33, 77, 78
Surgery, other than cardiovascular	02, 14, 20, 28, 49
Stress Test	T2B
Palliative care	17

© 2022 Cascino TM et al. *JAMA Network Open.*

eTable 3. Marginal Effects for 30-Day Readmissions Following Discharge From LVAD

Variable	Model 1	Model 2	Model 3	Model 4	Model 5	Model 6	Model 7	Model 8
Black race (vs. White)	0.008	0.006	0.007	0.003	0.000	0.001	0.001	0.001
	[-0.004, 0.021]	[-0.006, 0.019]	[0.020, -0.006]	[-0.013, 0.018]	[-0.017, 0.017]	[-0.017, 0.018]	[-0.024, 0.026]	[-0.025, 0.027]
Female (vs. Male)	0.005	0.004	0.004	0.004	0.004	0.004	-0.003	-0.003
	[-0.008, 0.018]	[-0.009, 0.017]	[-0.009, 0.017]	[-0.011, 0.020]	[-0.012, 0.019]	[-0.012, 0.019]	[-0.023, 0.017]	[-0.023, 0.017]
Survival, predicted		0.068	0.070	0.085	0.075	0.075	0.087	0.087
		[0.032, 0.103]	[0.034, 0.106]	[0.042, 0.128]	[0.030, 0.119]	[0.030, 0.119]	[0.023, 0.150]	[0.023, 0.150]
VAD propensity			0.022	0.031	0.027	0.027	0.009	0.009
			[-0.014, 0.058]	[-0.013, 0.075]	[-0.017, 0.071]	[-0.017, 0.071]	[-0.053, 0.070]	[-0.053, 0.070]
Patient age, per year					-0.0007	-0.0007	0.0002	0.0002
					[-0.001, -0.0001]	[-0.001, -0.0001]	[-0.001, 0.001]	[-0.001, 0.001]
Distance to LVAD Hospital, per 10 mi.						0.0002	0.000	0.000
						[-0.001, 0.001]	[-0.001, 0.001]	[-0.001, 0.001]
Low-income subsidy							0.021	0.021
							[-0.002, 0.043]	[-0.002, 0.043]
Social Deprivation Index								5.50e-05
								[-0.033, 0.034]
Observations	6,576	6,576	6,576	5,498	5,498	5,498	3,171	3,171
Hospital Fixed Effects	No	No	No	Yes	Yes	Yes	Yes	Yes
Year indicators	Yes	Yes	Yes	Yes	Yes	Yes	Yes	Yes
Part D enrollees only	No	No	No	No	No	No	Yes	Yes

Associations between race and gender with 30-day readmissions are shown in this table. Readmissions by race and gender were similar after adjusting for clinical risk (Model 2), LVAD propensity (Model 3), hospital fixed effect (Model 4), age (Model 5), distance to hospital (Model 6), LIS (Model 7), SDI (Model 8). Abbreviations: LVAD, left ventricular assist device; mi, miles.

© 2022 Cascino TM et al. *JAMA Network Open.*

eMethods 1. Sample Selection and LVAD Propensity Estimation

Initial sample. Our sample comprises Medicare FFS beneficiaries admitted to a hospital with a primary diagnosis of heart failure (ICD-9: 42830, 42831, 42832, and 42833 or receipt of LVAD) from 2008-2014. Our sample further required patients to have six months of eligibility preceding their hospital admission and residence within the continental United States, Hawaii, or Alaska.

Data challenges for sample selection. While heart failure is common, ventricular assist devices are only relevant for a small fraction of advanced heart failure (AHF) patients. Claims data generally, and ICD-9 codes, in particular, do not capture AHF and extant codes are measured with error. Diagnoses may be documented imprecisely (e.g., with three, as opposed to five digits) or not at all. These data do, however, capture many variables that are correlated with advanced heart failure such as repeated heart failure hospitalization as well as contraindications for LVAD implantation.

We thus employ a data-driven approach to selecting our analytic sample. We constructed a large dataset of patient-level variables that might be correlated with LVAD treatment. These variables are further described below. These data are drawn from the current hospitalization (demographics and diagnoses present on admission) as well as from inpatient and outpatient encounters in the preceding six months. The variables exclude provider-level factors and variables that could be a consequence of treatment decisions in index hospitalization as these could be endogenous. The variables also exclude patient race and sex as we do not wish to "control" for these factors when we measure disparities.

Empirical challenges. Our approach is similar to propensity score estimation, but we face two important challenges. First, we have a high-dimensional set of patient variables and an unknown functional form. We will address this issue using machine learning methods and cross-validation. Second, our data have a large class imbalance. Heart failure hospitalizations are prevalent (486,017 in our sample), but LVAD treatment is rare (7,135 in our sample). This imbalance exists because LVAD would be inappropriate for the vast majority of patients – who aren't relevant to our analysis – and because access may be limited by proximity and access to LVAD treatment centers. This large class imbalance illustrates the importance of selecting a relevant sample of non-LVAD patients, but it also poses a practical challenge for estimating our LVAD propensities – a model that predicted LVAD=0 for all observations would be 98.6% accurate (and 100% useless) in our sample. Furthermore, accurate prediction among HF patients with a low LVAD propensity is all but useless, we need to train a model that's accurate for patients with a non-trivial probability of receiving LVAD treatment.

Estimation strategy. We address the class imbalance using the synthetic minority oversampling technique (SMOTE). The SMOTE technique (Chawla et al., 2002) generates a synthetic sample that oversamples the rare outcome and undersamples the more prevalent outcome, to generate more accurate predictions in imbalanced data. We employed a 1:3 ratio for oversampling and undersampling. [1] We used eXtreme Gradient Boosting Training (XGBoost) algorithm (Chen and Guestrin 2016) to generate propensity scores with our synthetic sample. Cross-validation (10-fold) was used to select appropriate hyperparameters that maximized out-of-sample area under the ROC curve. This measure was chosen because it provides better discriminative power between the two values of the variable (between LVAD treatment and no LVAD treatment). Using the hyper-parameters chosen using the cross-validation, we then build a final XGBoost model for estimating the propensity scores. [2] The final model had an AUC of 0.9045.

Patients with a predicted probability of LVAD treatment ($\hat{V}$) of less than 0.05 were eliminated from our sample. This restricts our sample size to 15,076 observations with a common support for the LVAD propensity.

We further eliminated 19 observations with missing geographic data a further 13 observations resided in zip codes for which social deprivation data did not exist. Finally, we eliminated the 311 Hispanic patients in our sample.

---

[1] Model performance was not sensitive to alternative over/under sampling (e.g., 1:3-1:7), unless we ignored the imbalance entirely.

[2] Note that the XGBoost algorithms produce predictions, which we rescaled to propensities. This makes the VAD propensity marginal effects in Table 2, Models 3-5 more interpretable, but does not otherwise affect our results.

While this is an important and interesting population, the sample sizes were too small to calculate meaningful parameters.

Our analytic sample comprises 6,825 LVAD patients and 7,908 non-LVAD patients, a total of 14,733 observations.

Subsamples. Our analyses also examine subsamples using patient's low-income-subsidy (LIS) status and survival conditional upon LVAD receipt. LIS status is a beneficiary-specific measure of income and is only available for Medicare Part D beneficiaries. These data are only available for about 64% of our sample. In Table 2, Model 5, we lose 5372 observations. Two additional observations are lost in Table 2, Model 6. These observations are "present" in the SDI data, but the index value is missing.

The models reported in Table 3 are based on the 6,825 observations that received LVAD therapy. The sample size falls to 6,739 observations in Model 4 as we are limiting the model to within hospital variation. This produces collinearity problems for low-volume VAD centers. [3] As in Table 2, we lose 2,512 observations when the sample is restricted to Medicare Part D beneficiaries.

A. Severity Adjustment for LVAD and survival.

We predict one-year survival for each LVAD patient to measure severity. These predictions use the same independent variables used to estimate the LVAD propensity. The variables include diagnoses present on or in the six months preceding the index admission, prior utilization, and demographics excepting race and sex. The model is estimated using an XGBoost algorithm. SMOTE was not used as one-year survival among LVAD patients is reasonably balanced across outcome classes (i.e., N = 304, 796 with ≥ one-year survival and N=188,056 with < one-year survival). 10-fold cross-validation was employed to select hyperparameters of the XGBoost algorithm. The final model was then used to predict one-year survival for each LVAD patient. This model had an AUC of 0.94513.

[3] Note that we have estimated specifications of Models 5-8 without fixed effects and the findings are consistent with those reported in Table 3. The average marginal effects of race and sex are small and statistically insignificant.

© 2022 Cascino TM et al. *JAMA Network Open.*

eMethods 2. Empirical Methods

This section provides further detail regarding the estimation of models reported in Tables 2 and 3. All models are estimated via logistic regression. We report models from six specifications, but our conclusions regarding race and gender are robust to a variety of alternatives.

A. Table 2 Specifications

Model 1. Simply tests for differences in the probability of LVAD utilization by race and sex conditional upon the year of hospitalization. We allow for interactions between race=Black and sex=Female. Note that there are very few African American women in our sample and while we cannot reject the hypothesis that the African American Woman parameter is equal to zero, the data suggest that, ceteris paribus, African American women receive more aggressive LVAD treatment than African American men. Thus, our base specification is:

$$VAD = \alpha + Black\beta_{11} + Female\beta_{12} + Black * Female\beta_{12} + Year_t + \epsilon$$

where $Year_t$ is a set of year (2009 to 2014) fixed effects. All our models are estimated via logistic regression, but we describe them using a linear regression notation for ease of exposition.

Model 2 – LVAD propensity. We incorporate each patient's LVAD propensity, $\hat{V}$, as well as interactions between $\hat{V}$ and race and sex.

$$VAD = \alpha + f(Black, Female, \hat{V}; \beta_1) + Year_t + \epsilon$$

where $f(Black, Female, \hat{V}; \beta_1)$ includes a main effect for each variable as well as pairwise interactions between them, more formally: $Black\beta_{11} + Female\beta_{12} + Black * Female\beta_{13} + \hat{V}\beta_{14} + \hat{V} * Black\beta_{15} + \hat{V} * Female\beta_{16}$. These terms are included in all subsequent Table 2 models.

Model 3 – Age. The age patterns of cardiovascular disease differ across both sex and race. We condition on patient age and allow age effects to differ by race and sex through interaction terms. Heterogeneity in age effects by race and sex are described in Appendix Figure 1, below.

$$VAD = \alpha + f\left(Black, Female, \hat{V}; \beta_1\right) + Age\beta_2 + Black * Age\beta_3 + Female * Age\beta_4 + Year_t + \epsilon$$

Model 4 – Proximity. Access to LVAD therapy may differ geographically given the paucity of LVAD centers. We condition on the $Distance$ from a patient's zip code to the nearest VAD treatment center.

$$VAD = \alpha + f\left(Black, Female, \hat{V}; \beta_1\right) + Age\beta_2 + Black * Age\beta_3 + Female * Age\beta_4 + Distance\beta_5 + Year_t + \epsilon$$

Model 5 – LIS. The low-income subsidy from Medicare Part D is an individualized measure of poverty. We condition on LIS status among beneficiaries enrolled in both Medicare FFS (our main sample) and Medicare Part D.

$$VAD = \alpha + f\left(Black, Female, \hat{V}; \beta_1\right) + Age\beta_2 + Black * Age\beta_3 + Female * Age\beta_4 + Distance\beta_5 + LIS\beta_6 + Year_t + \epsilon$$

Model 6 – SDI. We also condition on the Social Deprivation Index (SDI) based on each patient's zip code. Table 2 reports marginal effects for the aggregated index, but results for race and sex were similar when using the disaggregated SDI components.

$$VAD = \alpha + f\left(Black, Female, \hat{V}; \beta_1\right) + Age\beta_2 + Black * Age\beta_3 + Female * Age\beta_4 + Distance\beta_5 + LIS\beta_6 + SDI\beta_7 + Year_t + \epsilon$$

Model 7 – Neighborhood random effects. We also allow for unobserved neighborhood-specific differences. We include random effects for each patients' five-digit zip code tabulation area.

$$VAD = \alpha + f\left(Black, Female, \hat{V}; \beta_1\right) + Age\beta_2 + Black * Age\beta_3 + Female * Age\beta_4 + Distance\beta_5 + LIS\beta_6 + SDI\beta_7 + Year_t + Neighborhood + \epsilon$$

© 2022 Cascino TM et al. *JAMA Network Open.*

B. Table 3 Specifications

Model 1. This model tests for differences in one-year survival by race and sex conditional upon the year of hospitalization. We allow for interactions between race=Black and sex=Female. Thus our base specification is:

$$Survival = \alpha + Black\beta_{11} + Female\beta_{12} + Black * Female\beta_{12} + Year_t + \epsilon$$

where $Year_t$ is a set of year (2009 to 2014) fixed effects.

Model 2 – Severity. We incorporate each patient's severity adjustment index, $\hat{S}$, as well as interactions between $\hat{S}$ and race and sex.

$$Survival = \alpha + f_1(Black, Female, \hat{S}; \beta_1) + Year_t + \epsilon$$

where $f_2(Black, Female, \hat{S}; \beta_1)$ includes a main effect for each variable as well as pairwise interactions between them, more formally: $Black\beta_{11} + Female\beta_{12} + Black * Female\beta_{13} + \hat{S}\beta_{14} + \hat{S} * Black\beta_{15} + \hat{S} * Female\beta_{16}$. These terms are included in all subsequent Table 3 models.

Model 3 – LVAD Propensity. This model conditions on patient's severity $(\hat{S})$ and LVAD propensity $(\hat{V})$. Each of these terms is separately interacted with race and sex, combining the terms from f and f_1 above.

$$Survival = \alpha + f_3(Black, Female, \hat{V}, \hat{S}; \beta_1) + Year_t + \epsilon$$

Model 4 – Hospital Fixed Effects. This model incorporates hospital fixed effects, h_i. This means that we are comparing survival across groups (i.e., white vs black, male vs female) within the same hospital. These terms would be particularly important if black patients were treated in hospitals with different unobserved quality levels or practice styles. We do find evidence that African American patients are not equally represented across hospitals, but conditioning on these unobserved hospital-level differences does not alter our findings.

$$Survival = \alpha + f_3(Black, Female, \hat{V}, \hat{S}; \beta_1) + h_i + Year_t + \epsilon$$

Note that we do not use hospital fixed effects in our LVAD utilization models (Table 2) as they would almost certainly introduce selection bias into that model. This is because LVAD insertion is only performed in a subset of hospitals – the choice of hospital may be a function of the LVAD decision.

Model 5 – Age. The age patterns of cardiovascular disease differ across both sex and race. We condition on patient age and allow age effects to differ by race and sex through interaction terms.

$$Survival = \alpha + f_3(Black, Female, \hat{V}, \hat{S}; \beta_1) + Age\beta_2 + Black * Age\beta_3 + Female * Age\beta_4 + h_i + Year_t + \epsilon$$

Model 6 – Proximity. Access to LVAD therapy may differ geographically given the paucity of LVAD centers. We condition on the $Distance$ from a patient's zip code to the nearest VAD treatment center.

$$Survival = \alpha + f_3(Black, Female, \hat{V}, \hat{S}; \beta_1) + Age\beta_2 + Black * Age\beta_3 + Female * Age\beta_4 + Distance\beta_5 + h_i + Year_t + \epsilon$$

Model 7 – LIS. The low-income subsidy (LIS) from Medicare Part D is an individualized measure of poverty. We condition on LIS status among beneficiaries enrolled in both Medicare FFS (our main sample) and Medicare Part D.

$$Survival = \alpha + f_3(Black, Female, \hat{V}, \hat{S}; \beta_1) + Age\beta_2 + Black * Age\beta_3 + Female * Age\beta_4 + Distance\beta_5 + LIS\beta_6 + h_i + Year_t + \epsilon$$

Model 8 – SDI. We also condition on the Social Deprivation Index (SDI) based on each patient's zip code. Table 2 reports marginal effects for the aggregated index, but results for race and sex were similar when using the disaggregated SDIcomponents.

© 2022 Cascino TM et al. *JAMA Network Open.*

$$Survival = \alpha + f_3(Black, Female, \hat{V}, \hat{S}; \beta_1) + Age\beta_2 + Black * Age\beta_3 + Female * Age\beta_4 + Distance\beta_5 + LIS\beta_6 + SDI\beta_7 + h_i + Year_t + \epsilon$$

Model 9 – Neighborhood random effects. We allow for unobserved neighborhood-specific differences. We include random effects for each patients' five-digit zip code tabulation area. These models exclude the hospital-specific fixed effects used in specifications 4-8.

$$Survival = \alpha + f_3(Black, Female, \hat{V}, \hat{S}; \beta_1) + Age\beta_2 + Black * Age\beta_3 + Female * Age\beta_4 + Distance\beta_5 + LIS\beta_6 + SDI\beta_7 + Year_t + Neighborhood + \epsilon$$

© 2022 Cascino TM et al. *JAMA Network Open.*

eFigure 1. Unconditional Effects of Race and Sex on LVAD Use

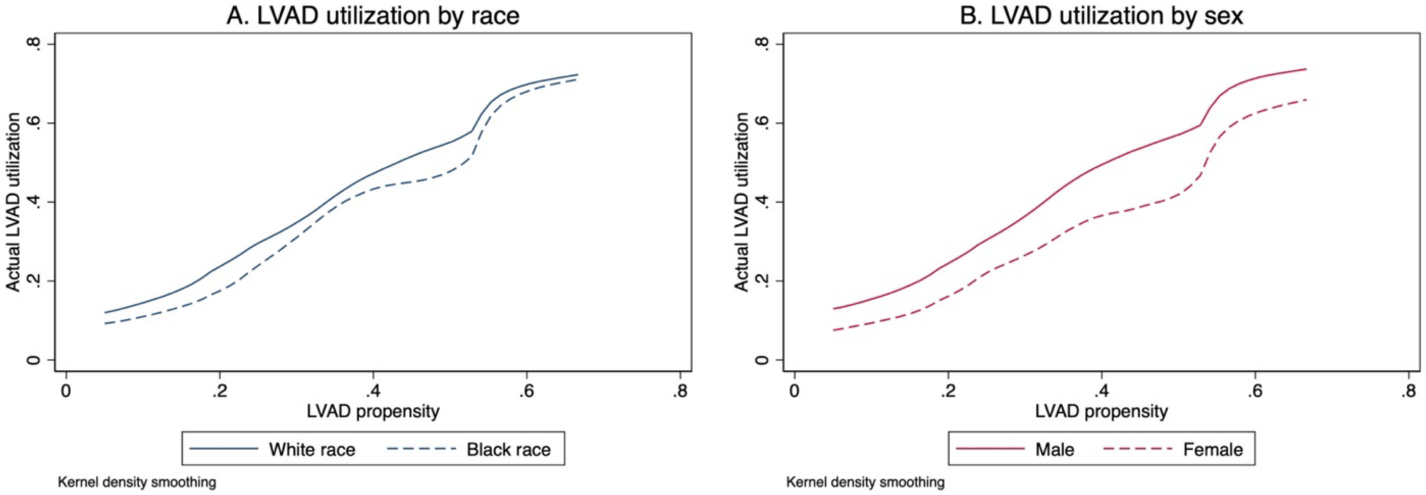

The unconditional effect of (A) race and (B) sex on the utilization of LVAD are shown.

© 2022 Cascino TM et al. *JAMA Network Open.*

eFigure 2. Marginal Effects of Black Race by Gender on LVAD Use

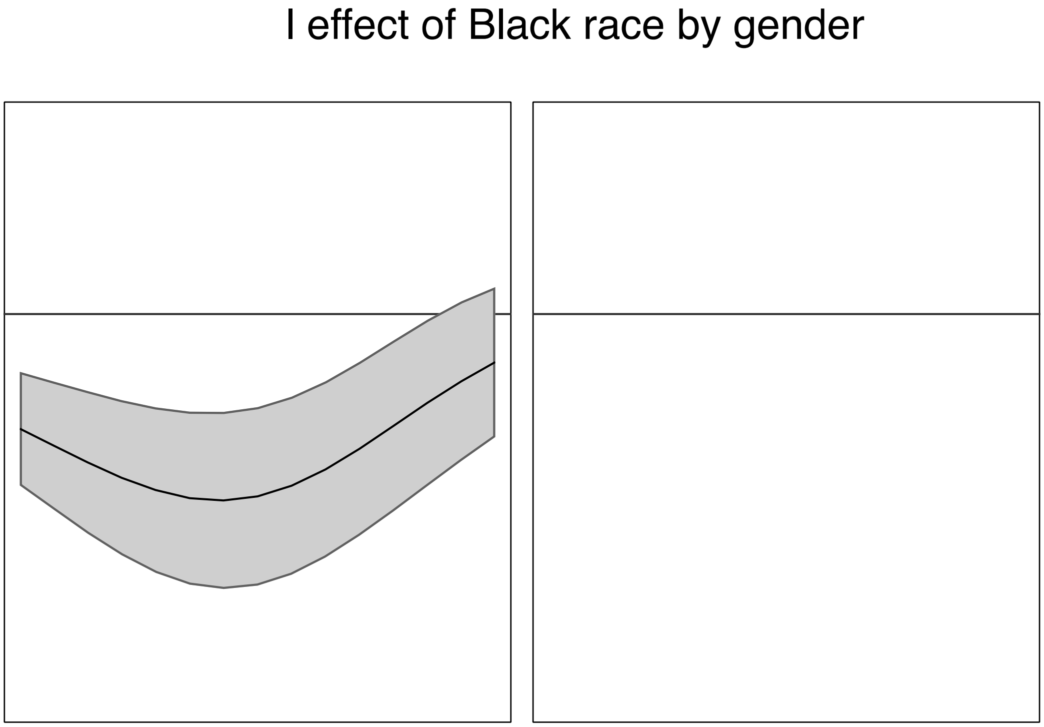

The marginal effect with 95% CIs of Black race among (A) men and (B) women on the utilization of LVAD conditional on clinical risk, age, distance to hospital, individual SES, and neighborhood effects (Model 6).

© 2022 Cascino TM et al. *JAMA Network Open.*

eFigure 3. Incremental Effect of Race and Gender on Survival by Expected Survival

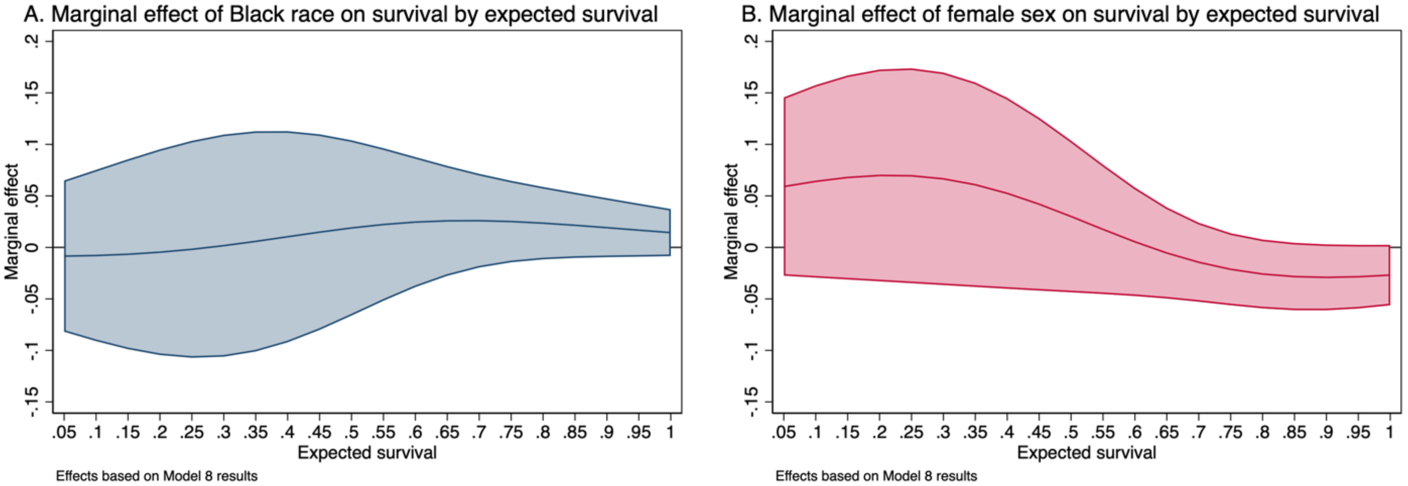

The posterior estimations of the marginal effect with 95% CIs of race and gender on one-year survival after LVAD adjusted for clinical risk, distance to hospital, individual SES, and neighborhood effects. There are no differences in survival by race (A) or gender (B) across the spectrum of expected survival.

© 2022 Cascino TM et al. *JAMA Network Open.*

References:

1. Chawla, N., Bowyer, K., Hall, L. and Kegelmeyer, W. 2002. SMOTE: Synthetic minority oversampling technique. Journal of Artificial Intelligence Research. 16, 321-357.

2. Chen, T., and Guestrin, C. "XGBoost: A Scalable Tree Boosting System," 22nd SIGKDD Conference on Knowledge Discovery and Data Mining, 2016

© 2022 Cascino TM et al. *JAMA Network Open.*
